# Fatigue is cross-sectionally not associated with objective assessments of inflammation, but changes in fatigue are associated with changes of disease activity assessments during biologic treatment of patients with established rheumatoid arthritis

**DOI:** 10.1007/s10067-020-05402-y

**Published:** 2020-10-11

**Authors:** Hilde Berner Hammer, Brigitte Michelsen, Joe Sexton, Till Uhlig, Sella A. Provan

**Affiliations:** 1 Department of Rheumatology, Diakonhjemmet Hospital, Box 23 Vinderen, N-0319 Oslo, Norway; 2 Faculty of Medicine, University of Oslo, Oslo, Norway; 3 Division of Rheumatology, Department of Medicine, Hospital of Southern Norway Trust, Kristiansand, Norway

**Keywords:** Fatigue, Rheumatoid arthritis, Ultrasound

## Abstract

**Objective:**

The associations between fatigue and disease activity in patients with rheumatoid arthritis (RA) have not been defined. The present objectives were to explore in RA patients the cross-sectional and longitudinal relation of fatigue with subjective as well as objective assessments of disease activity.

**Methods:**

RA patients were consecutively included when initiating biologic disease-modifying anti-rheumatic drugs (DMARDs) and assessed at baseline, 1, 2, 3, 6, and 12 months with investigation of fatigue, patient-reported outcome measures (PROMs; joint pain and patient’s global disease activity, MHAQ, pain catastrophizing, Mental Health score), clinical examinations (examiner’s global disease activity, 28 tender and swollen joint counts), and laboratory variables (ESR, CRP, calprotectin). Ultrasound examinations (semi-quantitative scoring (0–3)) with grey scale and power Doppler were performed of 36 joints and 4 tendons. Statistics included one-way analysis of variance, Pearson’s correlations, and multiple linear and logistic regression analysis.

**Results:**

A total of 208 RA patients (mean (SD) age 53.2 (13.2) years, disease duration 9.8 (8.5) years) were included. Fatigue levels diminished during follow-up (mean (SD) baseline/12 months; 4.8 (2.8)/3.0 (2.5) (*p* < 0.001)). Substantial correlations were cross-sectionally found between fatigue and PROMs (median (IQR) r=0.61 (0.52-0.71)) but not with the objective inflammatory assessments. During follow-up, baseline fatigue was associated with PROMs (*p* < 0.001) but not with objective inflammatory assessments. However, change of fatigue was associated with change in all variables. Higher baseline fatigue levels were associated with lower clinical composite score remission rates.

**Conclusion:**

Fatigue was cross-sectionally associated to subjective but not to objective disease assessments. However, change of fatigue during treatment was associated to all assessments of disease activity.

**Trial registration number:**

Anzctr.org.au identifier ACTRN12610000284066, Norwegian Regional Committee for Medical and Health Research Ethics South East reference number 2009/1254

**Table:** 

**Key Points** • *In this longitudinal study of patients with established RA, fatigue was associated with patient reported outcome measures at each visit, but not with objective assessments of inflammation including calprotectin and comprehensive ultrasound examinations*. • *Changes in fatigue during biological treatment were associated with changes in patient reported outcome measures, clinical, laboratory and ultrasound assessments*. • *Baseline fatigue was associated with all patient reported outcome measures, but not objective assessments of inflammation at all the prospective visits*. • *Higher baseline fatigue levels were associated with lower remission rates as assessed by clinical composite scores*.

## Introduction

Fatigue is a sensation of weakness, and lack of energy and severe fatigue has been found in more than 40% of patients with rheumatoid arthritis (RA) [1, 2], contributing to reduced health-related quality of life [3]. The following definition of chronic fatigue [4] may be used: “Fatigue is perceived as unpleasant, unusual, abnormal or excessive whole-body tiredness, disproportionate to or unrelated to activity or exertion and present for more than one month. Fatigue is constant or recurrent, it is not dispelled easily by sleep or rest and it can have profound negative impact on the person’s quality of life.” In a study exploring the importance patients placed on key RA-related symptoms found fatigue to be evaluated as the second most important outcome, only surpassed by pain [5]. Fatigue was subsequently included as one of seven patient-reported outcomes in the RA Impact of Disease (RAID) score, where all outcomes were rated on a numeric rating scale 0–10 [6], and fatigue is now considered a core domain to be measured and considered during RA flares and remission [7].

The pathophysiological background for fatigue in RA patients is not clear, and most studies indicate that fatigue has a multifactorial explanation, with association to both inflammatory and psychosocial factors. A recent review exploring predictors of fatigue in RA [8] described that factors such as pain, mental health, disability, and sleep were consistent predictors of fatigue, while the role of disease activity and inflammation seemed less clear. However, the authors commented on the lack of specific studies primarily designed to investigate the inflammatory biomarkers of fatigue and the need for future studies to determine the mechanisms of fatigue [8].

Several studies have shown that treatment with TNF blockade caused reduction of fatigue [9, 10], which indicates that cytokine-mediated mechanisms may be important in the fatigue pathogenesis. Moreover, the systemic inflammation in RA was found to be associated with activation of immunological mechanisms in the brain, and inflammation has been indicated as contributing to fatigue [11]. A recent study found patterns of connectivity on functional MRI that predicted fatigue, pain, and cognitive dysfunction in RA patients [12], suggesting that the level of inflammation may be associated with fatigue.

Since there is no agreement on whether fatigue is primarily associated with subjective or objective assessments of disease activity in RA, there is a need for studies exploring both the associations between fatigue and patient reported outcome measures (PROMs) as well as between fatigue and objective examinations of inflammation. Ultrasound is a sensitive imaging modality and is increasingly used to detect inflammation in RA patients and may assess the degree of synovitis [13–15]. In addition, the recently introduced inflammatory marker calprotectin (S100A8/S100A9 or MRP8/MRP14) is a major leukocyte protein shown to be superior to other inflammatory markers in reflecting the level of inflammation in patients with RA [16–18].

To capture different associations with fatigue, a longitudinal design with a homogenous group was chosen. Thus, we presently used a 1-year follow-up cohort of RA patients who initiated biologic disease-modifying anti-rheumatic drugs (bDMARDs). The objectives of our study were to explore the cross-sectional and longitudinal associations between the level of fatigue and PROMs as well as clinical, laboratory, and ultrasound assessments of disease activity.

## Patients and methods

### Patients

From a previously described cohort of 209 patients with established RA [19], 208 patients who had given information about their level of fatigue were presently included. The study (Anzctr.org.au identifier ACTRN12610000284066) was approved by the Norwegian Regional Committee for Medical and Health Research Ethics South East (reference number 2009/1254), and the patients gave their written informed consent according to the Declaration of Helsinki.

The length of education was noted, and the patients were divided into four groups depending on their highest obtained educational level according to the Norwegian system.

### Medication

All patients initiated a bDMARD when included in the study. This medication was continued during follow-up, and several bDMARDs were initiated: etanercept (35.1%), rituximab (20.2%), certolizumab (11.1%), infliximab (10.1%), tocilizumab (8.7%), adalimumab (6.7%), golimumab (5.3%), and abatacept (2.9%).

### Patient-reported outcome measures

The patients were examined when initiating bDMARDs and assessed at baseline and after 1, 2, 3, 6, and 12 months. The fatigue score (10 points numeric rating scale) from RAID was used to represent the level of fatigue.[20] Patients scored their joint pain (0–100 visual analogue scale (VAS)), patient’s global disease activity VAS (0–100), modified health assessment questionnaire (MHAQ, 0–3) [21], two main questions from the pain catastrophizing assessment [19], and short form-36 mental health scale score (SF36MH) [22].

### Clinical and laboratory assessments

The clinical examinations were performed by one of two highly experienced study nurses and included examiner’s global disease activity VAS (0-100) and 28 tender and swollen joint counts.

Laboratory assessments included erythrocyte sedimentation rate (ESR) (mm/h) and C-reactive protein (CRP) (mg/L) examined as part of the hospital routine laboratory examinations. In addition, calprotectin (μg/L) was measured in EDTA plasma with an ELISA from CALPRO AS (Lysaker, Norway) according to the instructions of the manufacturer [23].

The clinical composite disease activity scores were computed for each visit, and remission was calculated for each of the scores (Disease Activity Score (DAS) 28 (ESR) [24], Clinical Disease Activity Index (CDAI) [25]. and Simplified Disease Activity Index (SDAI) [26]). In addition, remission according to the ACR/EULAR Boolean remission criteria was calculated [27].

### Ultrasound assessments

An experienced sonographer (HBH) performed all the ultrasound examinations (using a Siemens Antares Excellence version, 5–13 MHz probe with PD frequency 7.3 MHz and PRF 391 Hz, using the same machine with no updates of the software during the study), blinded from the clinical assessments and laboratory markers from the same time points, as well as from previous ultrasound (US) results. Grey scale (GS) ultrasound reflecting synovial hypertrophy and power Doppler (PD) ultrasound reflecting vascularity in the synovium were scored as previously described [28] by use of a semi-quantitatively scale (0 = no, 1 = minor, 2 = moderate, 3 = major presence) of 36 joints (bilateral wrist (radiocarpal, midcarpal, radioulnar joints scored separately), metacarpophalangeal 1-5, proximal interphalangeal 2-3, elbow, knee, ankle (tibiotalar), metatarsophalangeals 1-5) with the Norwegian US atlas as reference [28], as well as four tendon sheaths (bilateral extensor carpi ulnaris and tibialis posterior). The sonographer has previously shown high reliability for US assessments of these joints and tendons [28, 29]. At each visit the sum scores of GS as well as PD were calculated and used as the ultrasound results.

### Statistics

Pearson’s correlation coefficients were used to assess bivariate associations. Correlation coefficients were defined as no < 0.2, low 0.2–0.3, moderate > 0.3–< 0.5, substantial 0.5–0.7, and > 0.7 high associations. Analysis of variance (ANOVA) was used to explore the associations between baseline PROMs as well as clinical, laboratory, and ultrasound assessments across quartiles of baseline fatigue levels (range 0–10, and divided into four groups depending on scores 0–2, 3–4, 5–7, or 8–10), and ANOVA was also used to explore the associations between the length of education and baseline PROMs, clinical, laboratory, and ultrasound assessments. In addition, the definition of fatigue being present was defined by scores ≥ 4. The associations between baseline fatigue and PROMs, clinical, laboratory, and ultrasound assessments at subsequent visits were explored in linear regression models adjusted for age and gender. Associations between changes in fatigue and changes in PROMs, clinical, laboratory, and ultrasound assessments were explored by use of linear regression models. Change was defined relative to baseline, with the change in fatigue treated as the dependent variable.

Remission defined as no swollen joints or sum score power Doppler of 0, 1, 2, or 3 was also explored. The predictive value of fatigue on remission at 6 and 12 months was explored by use of binary logistic regression, adjusted for age, gender, and disease duration.

Several analyses were presently included, but multiple comparison adjustments were not performed. Last observation carried forward replaced missing data (< 5% of the different variables). All calculations were performed by use of SPSS Statistics version 21 or STATA 16, and *p* < 0.05 was considered statistically significant.

## Results

The mean (SD) age of the 208 patients was 53.2 (13.2) years and disease duration 9.8 (8.5) years (81.3% were women, 79.6% anti-CCP positive, 60.0% rheumatoid factor positive). For several reasons, but mostly because of intolerance or lack of efficacy of the bDMARD, some patients dropped out of the study, and the number of patients was 208 (100%) at 1 month, 204 (98%) at 2 months, 197 (95%) at 3 months, 183 (88%) at 6 months, and 160 (77%) at 12 months.

Information on length of education was available in 204 patients with the following distribution: 9 years in 19 patients (11.3%), 12 years in 71 patients (34.3%), 16 years in 54 patients (26.5%), and more than 16 years in 57 patients (27.9%).

### Baseline

#### Fatigue levels

Mean (SD) fatigue level was at baseline 4.8 (2.8), and the fatigue scores were distributed across the full range of scores.

#### Associations with length of education

With increasing length of education, the baseline fatigue level was lower (*p* = 0.048). Also, all baseline PROMs were lower with increasing education (patient’s global VAS, *p* = 0.012; joint pain, *p* < 0.001; MHAQ, *p* = 0.001; tender joint count, *p* = 0.034; pain catastrophizing *p* = 0.006), while no associations between length of education and examinator’s global VAS, swollen joint count, laboratory markers, or ultrasound assessments were found.

#### Associations across quartiles of fatigue

**Fig. 1:**
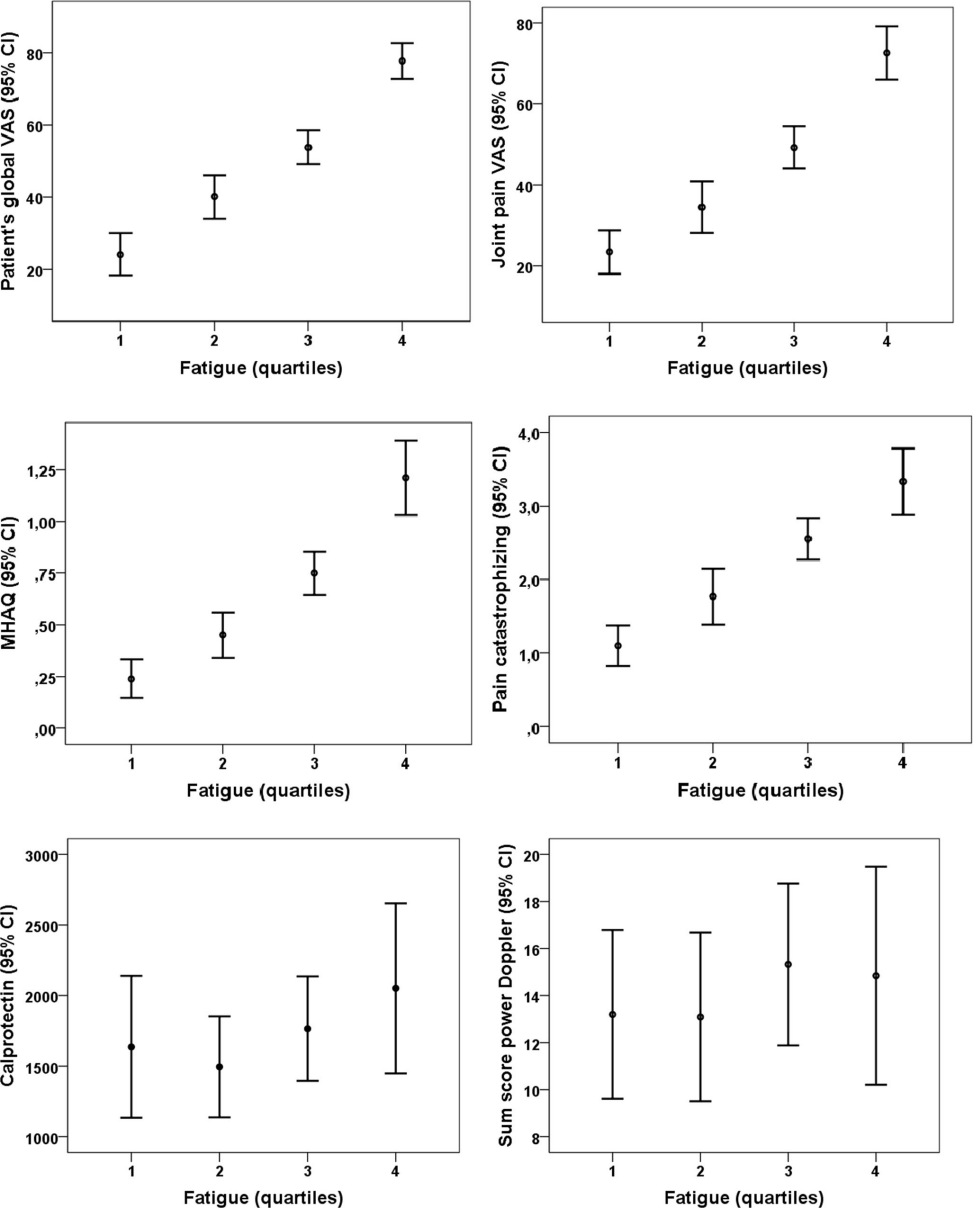
Mean (95% confidence interval) of patient reported outcome measures, calprotectin and sum score p ower Doppler at baseline across quartiles of baseline fatigue (error bar plots)

There were significant increases of all baseline PROMs across quartiles of baseline fatigue (*p* < 0.001), while there were no significant associations for CRP, calprotectin or sum scores GS and PD (see Fig. 1).

### Follow-up

#### Changes in fatigue, PROMs, clinical, laboratory, and ultrasound variables

Fatigue, as well as all PROMs, clinical, laboratory, and ultrasound assessments decreased significantly from baseline during follow-up (*p* ≤ 0.002 for all); Table 1 shows the mean (SD) of all variables. The median (IQR) of fatigue was at 1 month 3.0 (1.25–6.75), at 2 months 3.0 (1.0–5.0), at 3 months 3.0 (1.0–4.0), at 6 months 2.0 (1.0–5.0), and at 12 months 2.0 (1.0–5.0). Most patients improved their fatigue scores from baseline to 12 months (102 patients (63.8%), median (IQR) improvement 2 (1 to 4)), while 33 patients (20.6%) had unchanged scores and 25 patients (15.6%) had increased fatigue scores (median (IQR) − 1 (− 1 to − 2.5).

**Table 1:** Changes in patient reported outcomes, clinical, laboratory and ultrasound measures of disease activity over 12 months in patients with rheumatoid arthritis initiating biologic DMARDs

	Baseline	1 month	2 months	3 months	6 months	12 months
Variables (range)	*n* = 208	*n* = 208	*n* = 204	*n* = 197	*n* = 183	*n* = 160
Fatigue VAS (0–10)	4.8 (2.8)	3.9 (2.9)	3.4 (2.7)	3.0 (2.5)	2.9 (2.5)	3.0 (2.5)
PGA (0–100)	48.9 (26.8)	33.4 (26.6)	28.8 (24.7)	26.0 (23.1)	24.7 (22.9)	24.6 (22.4)*
Joint pain VAS (0–100)	44.9 (27.0)	30.9 (26.4)	26.4 (24.2)	23.3 (22.9)	21.8 (21.8)	22.1 (22.3)*
MHAQ (0–3)	0.66 (0.56)	0.47 (0.48)	0.47 (0.99)	0.38 (0.42)	0.38 (0.47)	0.38 (0.47)*
Examiner’s global VAS (0–100)	30.0 (15.6)	23.2 (15.3)	20.1 (13.3)	17.4 (12.8)	16.2 (11.8)	15.5 (12.4)*
Tender joints (of 28)	5.8 (6.1)	4.9 (5.6)	4.3 (5.6)	3.9 (5.6)	2.9 (4.8)	2.7 (4.7)*
Swollen joins (of 28)	6.3 (5.2)	5.1 (4.9)	4.7 (4.9)	4.1 (4.6)	3.4 (4.4)	2.9 (4.3)*
SF36 Mental Health	70.8 (20.8)	73.7 (20.9)	74.8 (19.9)	76.8 (19.6)	78.3 (18.8)	78.2 (17.6)
Pain catastrophizing score (0–6)	2.2 (1.5)	1.9 (1.4)	1.7 (1.4)	1.6 (1.4)	1.5 (1.5)	1.4 (1.3)*
DAS28 (0–10)	4.5 (1.5)	3.9 (1.5)	3.6 (1.5)	3.6 (1.5)	3.1 (1.4)	3.1 (1.3)*
CDAI (0–76)	20.0 (11.9)	15.7 (11.7)	13.8 (11.2)	12.4 (11.0)	10.4 (9.9)	9.6 (9.5)*
SDAI (0–86)	21.3 (12.5)	16.6 (12.2)	14.4 (11.7)	13.0 (11.3)	10.8 (10.2)	10.1 (9.9)*
ESR (mm/h)	28.1 (21.7)	22.5 (20.7)	20.4 (18.7)	19.5 (17.7)	17.5 (14.9)	17.1 (14.2)*
CRP (mg/L)	12.9 (18.9)	8.6 (15.4)	6.8 (13.2)	5.6 (11.4)	4.7 (9.0)	4.6 (11.5)*
Calprotectin (μg/L)	1740 (1629)	1182 (1334)	1016 (1035)	1019 (1194)	866 (769)	874 (823)
Sum score GS (0–120)	30.1 (18.8)	26.4 (17.3)	25.1 (17.4)	22.3 (15.3)	21.3 (14.9)	19.5 (14.3)
Sum score PD (0–120)	14.3 (13.6)	11.1 (12.0)	10.1 (11.6)	8.8 (10.7)	7.7 (10.1)	6.0 (8.1)

*n = 152

*PGA* patient’s global assessment of disease, *VAS* visual analogue scale (0–100), *MHAQ* Modified Health Assessment Questionnaire, *SF36 Mental Health* short form-36 mental health scale score, *DAS28* Disease Activity Score based on 28 joints and ESR, *CDAI* Clinical Disease Activity Index, *SDAI* Simplified Disease Activity Index, *ESR* Erythrocyte Sedimentation Rate, *CRP* C-reactive protein, *GS* grey scale ultrasound, *PD* power Doppler ultrasound

#### Differences between groups dependent on presence of fatigue

At baseline, patients with fatigue (score ≥ 4) versus (vs) no fatigue (score ≤ 3) had significantly higher mean (SD) DAS28 (5.1 (1.4) vs 3.6 (1.2)), CDAI (23.7 (12.0) vs 13.3 (8.2)), SDAI (25.1 (12.6) vs 14.4 (8.9)), patient’s global VAS (60.1 (22.7) vs 28.8 (21.4)), joint pain VAS (54.5 (25.5) vs 27.6 (20.2)), MHAQ (0.87 (0.55) vs 0.28 (0.32)), pain catastrophizing (2.7 (1.4) vs 1.2 (1.0)), and lower SF36 Mental Health scale score (63.3 (20.8) vs 84.3 (12.1)) (*p* < 0.001 for all variables), while there were no significant differences between the groups for calprotectin or sum scores of GS or PD. During follow-up, similar levels of difference between patients with versus without fatigue was found for the clinical composite scores as well as for the PROMs (*p* ≤ 0.001), while there were no significant differences for calprotectin or ultrasound, as well as except for one visit, also, no differences between the groups for CRP or swollen joint count.

#### Correlations between fatigue and subjective/objective variables

Table 2 gives the cross-sectional correlations between fatigue and PROMs, clinical, laboratory, and ultrasound assessments at each visit. During the study there were substantial to high correlations between fatigue scores and patient’s global VAS, joint pain VAS, MHAQ, SF36 Mental Health scale score, pain catastrophizing, and tender joint count. Only low correlations were seen between fatigue and examiner’s global VAS, and no correlations between fatigue and calprotectin, CRP, swollen joint count, or ultrasound findings.

**Table 2:** Cross-sectional correlations between fatigue and patient reported, clinical, laboratory and ultrasound variables at all visits in patients with rheumatoid arthritis initiating biologic DMARDs.

	Baseline, *n* = 208	1 month, *n* = 208	2 months, *n* = 204	3 months, *n* = 197	6 months, *n* = 183	12 months, *n* = 160
Patient’s global VAS	0.73**	0.76**	0.70**	0.70**	0.76**	0.66**
Joint pain VAS	0.64**	0.70**	0.65**	0.63**	0.71**	0.47**
MHAQ	0.62**	0.58**	0.22*	0.48**	0.62**	0.41**
SF36 Mental Health	− 0.56**	− 0.67**	− 0.55**	− 0.58**	− 0.61**	− 0.55**
Pain catastrophizing	0.58**	0.60**	0.52**	0.52**	0.50**	0.42**
28 tender joint count	0.48**	0.50**	0.47**	0.51**	0.54**	0.26*
Examiner’s global VAS	0.28**	0.27**	0.23*	0.29**	0.32**	0.17*
Calprotectin (μg/L)	0.07	0.14*	0.08	0.02	0.18*	0.11
CRP (mg/L)	0.11	0.13	0.09	0.09	0.14	0.02
28 swollen joint count	0.15*	0.08	0.09	0.09	0.10	0.01
Sum score GS	0.02	0.03	0.02	0.02	0.02	0.05
Sum score PD	0.03	0.05	0.01	0.03	0.02	0.01

*VAS* visual analogue scale, *MHAQ* modified health assessment questionnaire, *SF36 Mental Health* short form 36 Mental Health scale score, *CRP* C-reactive protein, *GS* grey scale ultrasound, *PD* power Doppler ultrasound

*p < 0.05, **p < 0.001

#### Prediction of baseline fatigue on follow-up subjective/objective variables

Baseline fatigue significantly predicted PROMs during follow-up, with associations having similar levels of significance at one as well as at 12 months. However, there were no significant associations between baseline fatigue and swollen joint count, laboratory variables, or sum score GS and PD ultrasound (Table 3).

**Table 3:** Baseline fatigue as a predictor of patient reported, clinical, laboratory and ultrasound assessments at prospective visits for patients with rheumatoid arthritis initiating biologic DMARDs

Dependent variables	1 month, *β* (95% CI)	2 months, *β* (95% CI)	3 months, *β* (95% CI)	6 months, *β* (95% CI)	12 months, *β* (95% CI)
Patient’s global VAS	5.64** (4.59–6.69)	4.77** (3.73–5.81)	3.83** (2.79–4.86)	4.16** (3.13–5.20)	3.83** (2.69–4.98)
Joint pain VAS	5.28** (4.20–6.35)	4.17** (3.12–5.23)	3.47** (2.43–4.52)	3.69** (2.67–4.71)	3.42** (2.25–4.58)
MHAQ	0.09** (0.07–0.11)	0.07** (0.05–0.09)	0.06** (0.04–0.08)	0.08** (0.05–0.10)	0.08** (0.05–0.10)
SF36 Mental Health	− 4.19** (− 5.06 to − 3.21)	− 3.20** (− 4.10 to − 2.30)	− 3.23** (− 4.14 to − 2.33)	− 3.53** (− 4.40–2.67)	− 2.96** (− 3.86 to − 207)
Pain catastrophizing	0.28** (0.21–0.34)	0.25** (0.18–0.32)	0.21** (0.14–0.28)	0.22** (0.14–0.29)	0.20** (0.13–0.27)
28 tender joint count	0.88** (0.63–1.12)	0.73** (0.48–0.99)	0.81** (0.56–1.07)	0.64** (0.41–0.87)	0.51** (0.25–0.77)
Examiner’s global VAS	1.08* (0.36–1.81)	0.72* (0.07–1.36)	1.02* (0.40–1.64)	1.07** (0.50–1.65)	0.84* (0.15–1.54)
Calprotectin (μg/L)	0.04 (− 0.02–0.11)	0.04 (− 0.01–0.09)	0.02 (− 0.04–0.08)	0.04 (− 0.00–0.08)	0.04 (− 0.01–0.08)
CRP (mg/L)	0.52 (− 0.24–1.27)	0.40 (− 0.26–1.06)	0.20 (− 0.39–0.78)	0.49* (0.02–0.97)	0.14 (− 0.53–0.80)
28 swollen joint count	0.08 (− 0.16–0.32)	0.11 (− 0.12–0.35)	0.16 (− 0.07–0.39)	0.13 (− 0.10–0.35)	0.09 (− 0.16–0.33)
Sum score GS	− 0.11 (− 0.98–0.75)	− 0.40 (− 1.31–0.50)	− 0.35 (− 1.13–0.44)	− 0.50 (− 1.27–0.26)	− 0.64 (− 1.37–0.09)
Sum score PD	− 0.00 (− 0.55–0.54)	− 0.08 (− 0.64–0.47)	− 0.00 (− 0.52–0.52)	− 0.10 (− 0.58–0.38)	− 0.29 (− 0.65–0.07)

Linear regression models were performed with either of PROMs, clinical, laboratory or ultrasound assessments as the dependent variable. *β* coefficients are shown for baseline fatigue, and the models are adjusted for age and gender

*VAS* visual analogue scale, *MHAQ* modified health assessment questionnaire, *SF36 Mental Health* short form 36 Mental Health scale score, *DAS28* disease activity score with 28 joints and erythrocyte sedimentation rate, *CDAI* clinical disease activity score, *SDAI* simple disease activity score, *CRP* C-reactive protein, *GS* grey scale ultrasound, *PD* power Doppler ultrasound

*p < 0.05, **p < 0.001

#### Associations between change of fatigue and subjective/objective variables

The regression coefficient for change in fatigue compared with change in PROMs, clinical, laboratory, and ultrasound assessments during the study showed significant associations between the change in fatigue and all subjective as well as objective variables (Table 4).

**Table 4:** Regression coefficient for change in patient reported, clinical, laboratory and ultrasound assessments in relation to change of fatigue during 1-year follow-up of patients with rheumatoid arthritis initiating biologic DMARDs.

	1 month	2 months	3 months	6 months	12 months
Patients global	1.05** (0.84,1.26)	1.04** (0.79,1.29)	1.12** (0.86,1.39)	0.93** (0.67,1.18)	0.8** (0.48,1.12)
Joint pain VAS	0.47* (0.17,0.76)	0.51* (0.19,0.82)	0.59** (0.28,0.91)	0.36* (0.05,0.66)	0.33 (− 0.01,0.68)
MHAQ	0.54** (0.29,0.79)	0.25 (− 0.03,0.52)	0.47* (0.16,0.77)	0.68** (0.4,0.95)	0.56* (0.23,0.89)
SF36 Mental Health	− 0.77** (− 1, − 0.54)	− 0.78** (− 1.04, − 0.53)	− 0.7** (− 0.98, − 0.43)	− 0.53** (− 0.79, − 0.27)	− 0.35* (− 0.66, − 0.04)
Pain Catastrophizing	0.62** (0.38,0.86)	0.63** (0.36,0.89)	0.79** (0.52,1.06)	0.66** (0.4,0.92)	0.43* (0.1,0.76)
Tender joint count	0.36* (0.11,0.61)	0.34* (0.06,0.62)	0.4* (0.12,0.69)	0.35* (0.08,0.62)	0.16 (− 0.18,0.49)
Examiner’s global VAS	0.61** (0.37,0.85)	0.64** (0.37,0.91)	0.52** (0.24,0.8)	0.62** (0.36,0.88)	− 0.11 (− 0.45,0.22)
Calprotectin	0.45** (0.2,0.7)	0.52** (0.25,0.79)	0.37* (0.09,0.66)	0.47** (0.21,0.74)	0.58** (0.29,0.87)
CRP	0.12 (− 0.13,0.38)	0.34* (0.07,0.61)	0.4* (0.12,0.69)	0.23 (− 0.04,0.5)	0.4* (0.08,0.72)
Swollen joint count	0.33* (0.08,0.58)	0.26 (− 0.01,0.54)	0.22 (− 0.07,0.51)	0.36* (0.1,0.63)	0.45* (0.13,0.77)
Grey Scale	0.33* (0.08,0.58)	0.35* (0.07,0.62)	0.55** (0.28,0.83)	0.43* (0.17,0.69)	0.29 (− 0.01,0.6)
Power Doppler	0.27* (0.01,0.52)	0.35* (0.08,0.62)	0.49** (0.21,0.77)	0.43* (0.17,0.69)	0.41* (0.11,0.72)

Table gives regression coefficient for (standardized) change in patient reported, clinical, laboratory and ultrasound assessments (independent variable) and change in fatigue (dependent variable). All values are adjusted for baseline fatigue, with 95% CI in parentheses

*VAS* visual analogue scale, *MHAQ* modified health assessment questionnaire, *SF36 Mental Health* short form 36 Mental Health scale score, *DAS28* disease activity score with 28 joints and erythrocyte sedimentation rate, *CDAI* clinical disease activity score, *SDAI* simple disease activity score, *CRP* C-reactive protein, *GS* grey scale ultrasound, *PD* power Doppler ultrasound

*p < 0.05, **p < 0.001

#### Prediction of baseline fatigue on achieving remission at 6 and 12 months

Baseline fatigue level predicted a reduced achievement of DAS28, CDAI, SDAI, and Boolean remission at 6 and 12 months (Table 5). However, baseline fatigue did not predict clinical remission assessed as no swollen joints or sum score PD ultrasound of zero (as well as when explored for sum scores PD of 1, 2, or 3).

**Table 5:** Logistic regression analyses for baseline fatigue as predicting clinical composite score remission

6 months	Odd ratios (CI)	12 months	Odd ratios (CI)
DAS28	0.80 (0.71, 0.91) (*p* = 0.001)	DAS28	0.80 (0.70, 0.91) (*p* = 0.001)
CDAI	0.78 (0.67, 0.91) (*p* = 0.002)	CDAI	0.70 (0.58, 0.83) (*p* < 0.001)
SDAI	0.82 (0.71, 0.95) (*p* = 0.006)	SDAI	0.76 (0.65, 0.89) (*p* = 0.001)
Boolean	0.86 (0.75, 0.99) (*p* = 0.039)	Boolean	0.78 (0.66, 0.92) (*p* = 0.003)

Logistic regression analyses with DAS28, CDAI, SDAI or Boolean remission at 6 and 12 months as dependent variables with baseline fatigue, gender, age and disease duration as independent variables. Odd ratios are given with 95% confidence interval (CI)

## Discussion

In this longitudinal observational study on patients with established RA there were no consistent associations in cross-sectional assessments during follow-up between fatigue and objective assessments of inflammation, while there were substantial to high associations between fatigue and all the different PROMs. However, change of fatigue was significantly associated with change in all the subjective as well as objective assessments. In addition, baseline fatigue was found to be a negative predictor of achieving clinical composite score remission.

The level of fatigue was negatively associated with the length of education. This supports previous studies [30] and may have several reasons, including potentially more manual work as well as shift work, which has been shown to increase fatigue [31].

In the study by Druce et al [10], RA patients reported substantial improvement in their fatigue after commencing anti-TNF-alpha therapy. The minimal important difference (MID) for a single item fatigue question by use of VAS 0–10 was found to be ranged between 0.8 and 1.1 for improvement [32]. Compared with this MID, we found that most of our patients reached MID after initiating bDMARD treatment, with improvement seen already after one month and with a substantial improvement during the study.

Fatigue was presently found to be highly associated to PROMs, and PROMs are important parts of the clinical composite scores like DAS28, CDAI, and SDAI. Thus, the clinical composite scores may be influenced by the levels of fatigue. Increased clinical composite scores may lead to intensified medical treatment. Some studies have shown that RA patients with fibromyalgia are treated more aggressively with bDMARDs than RA patients without fibromyalgia because of higher clinical composite scores [33]. The present finding of reduced achievement of clinical composite score remission with increasing baseline fatigue supports other studies [34] urging an awareness of subjective causes and not inflammation to be a potential cause of elevated composite scores [35]. Thus, it is of major importance to treat fatigue and other PROMs that are not associated to inflammation with other alternative approaches than to increase the medication.

The functional MRI study that indicated RA inflammation to predict fatigue, pain, and cognitive dysfunction used DAS28 as a measure for inflammatory activity [12]. This composite score includes tender joints given double weight compared with swollen joints, and it includes patient’s global VAS [26]. Our study finds fatigue to be highly associated to both of these two PROMs, and thus, the functional MRI study could have been influenced by subjective assessments driving the disease activity as measured by DAS28, and it may thus not have been objective inflammation predicting fatigue.

In a study of two early RA cohorts receiving standard or treat-to-target treatment, most patients improved function, while the groups with a less favorable outcome in HAQ were found to have more fatigue at baseline [36], suggesting that such patients may benefit from therapies targeted at improving function in addition to those targeted at suppression of inflammation. The present study as well as other studies [37] supports this finding, with baseline fatigue being highly associated with function as assessed by MHAQ during follow-up.

Fatigue increases the burden of disease in patients with RA [1, 5]. A study of early RA patients found severe fatigue in about half of the cohort [38], and despite a strict treat-to-target strategy, fatigue remained an overall problem during the study. In a study by van Hoogmoed et al [1], 42% of established RA patients were found to have severe fatigue. As in our study, they found psychosocial factors, rather than inflammation-related factors, to play an important role in fatigue severity in RA. A Cochrane review on RA patients treated with bDMARDs found a small to moderate improvement in fatigue [39]. However, the evaluation was that it is unclear whether the improvement results from a direct action of the biologics on fatigue or indirectly through reduction in inflammation, disease activity, or some other mechanism. A recent study explored the relationship between personality traits and fatigue in RA patients and found depression and disability to be the major correlates of fatigue [40]. This is supported by our study, where both the SF36 Mental Health and MHAQ were found to have substantial correlations with fatigue during follow-up.

In our study we found change of fatigue during the study to be associated with PROMs as well as clinical, laboratory, and ultrasound assessments. Thus, when bDMARD treatment causes reduction of inflammation, the fatigue is also reduced. The lack of associations between fatigue and the inflammatory assessments at each of the visits, but associations between the change of fatigue and inflammatory variables, indicates that fatigue is responding to improvement of inflammation, even if it is not associated with the level of the inflammatory variables cross-sectionally. We presently included calprotectin and comprehensive ultrasound assessments to obtain more sensitive measures of inflammation. Thus, our study may support other studies where the decreased fatigue during anti-rheumatic treatment was evaluated not to be directly caused by the reduced inflammation [2, 41].

A weakness of our study is that we only have one question regarding fatigue. However, the fatigue question was part of the RAID questionnaire which has been developed by patients and should thus be relevant for exploring fatigue. In addition, a numeric rating scale of 0–10 (as also used for RAID fatigue) has been validated for fatigue in psoriatic arthritis [42]. Another potential weakness is that this is a single-centre study, which may reduce the generalizability of our findings. On the other hand, this approach may increase the reliability of the different clinical examinations and could therefore strengthen our results. It may be argued that we should have included a control group. However, the objectives of this study were to explore to which extent fatigue was associated with objective measures of inflammation. Thus, the present inclusion of a high number of RA patients initiating bDMARD supposed to be effective for controlling inflammation should be a valuable group to explore the associations between fatigue and subjective as well as objective assessments of disease activity. Of importance is the inclusion only of patients with long-lasting RA. Thus, the present findings are only representative for established RA patients, since the new recommendation of aggressive treatment of early RA [43] may result in different groups having fatigue. Further studies should explore the cause of fatigue in RA patients with different disease durations.

In conclusion, this longitudinal observational study of patients with established RA found no or low cross-sectional associations between the levels of fatigue and inflammation as assessed by sensitive objective measures including calprotectin and ultrasound, while fatigue was strongly associated with all the different PROMs. Fatigue levels declined during bDMARD therapy, with changes associated with those of PROMs and objective assessments. Thus, improving inflammation during bDMARD treatment caused a reduction of fatigue in patients with established RA even if it was unrelated to the assessed degree of inflammatory activity.

## Data Availability

Upon reasonable request the authors are be prepared to send relevant documentation or data
